# Supplementary material for: Self-management of type 2 diabetes mellitus in pregnancy and breastfeeding experiences among women in Thailand: Study protocol
Source: PLoS One. 2023 Jun 12;18(6):e0286646. doi: 10.1371/journal.pone.0286646 (PMC10259789; doi:10.1371/journal.pone.0286646)
Supplement: S1 File — (DOCX) [file pone.0286646.s001.docx]

**SUPPLEMENT 1** Interview guide

**Pregnancy Appointment (T1)**

| **Interview Questions (*Probing Questions in Italic*)** | |
| --- | --- |
| **Aim 1: Attitudes and confidence toward diabetes self-management** |  |
| 1. How has having diabetes affected you? *How did you feel about it?* | Attitude |
| 2. Tell me about how you managed your diabetes before you became pregnant? *Tell me about your confidence in managing your diabetes?* | Confidence |
| 3. How has having diabetes in pregnancy affected you? *How did you feel about it?* | Attitude |
| 4. Tell me about how you are now managing your diabetes since you became pregnant? *Tell me about your confidence in managing your diabetes?* | Confidence |
| **Aim 2: Barriers and facilitators of diabetes self-management in pregnancy** |  |
| 4. Please share with me what you eat and how you are physically active during your pregnancy? *Can you give me examples of the food you eat? Can you give me examples of your daily physical activity?* | General diabetes self-management |
| 5. What have you found to be hard (barriers) in managing your diabetes during pregnancy? *Can you give me some examples? What happened then?* | Barriers |
| 6. What have you found to be helpful (facilitators) when managing your diabetes? *Can you give me some examples? What happened then?* | Facilitators |
| 7. Can you please share what education you have received to help you manage your diabetes? Have you received additional education regarding diabetes self-management since you became pregnant? *Is there anything that you think you need more information on?* | Facilitators |
| **Aim 3: Breastfeeding confidence and intention in pregnancy** |  |
| 8. What does breastfeeding mean to you? | N/A |
| 9. Describe for me, if you can, any influence having diabetes might have on your plan to breastfeed your baby. | Confidence |
| 10. Share with me, if you can, how you came to the decision to breastfeed your baby.  **If you have had prior breastfeeding experiences, how were they?* | Intention |
| 11. Is there anything else you would like to share or any other questions you may have? Or Is there anything you may not have thought about related to the questions from the interview? I look forward to talking with you next time. | N/A |

*Note.* N/A= not applicable; * = probing questions for multigravida pregnant women

**Thank you for your time and thoughtful responses.**

**Postpartum Appointment (T2)**

| **Interview Questions (*Probing Questions in Italic*)** | |
| --- | --- |
| **Aim 4: Breastfeeding barriers and facilitators** |  |
| 1. How was your experience giving birth at the hospital? *Can you tell me about breastfeeding support that you received during your hospital stay?* | Facilitators  or Barriers |
| 2. What factors influenced your decision on how to feed your baby? *Can you give me some examples?* | Facilitators  or Barriers |
| 3. Was there anyone in your life that helped you come to that decision: such as your family members, spouse, friends, health care providers, or community? **If you had prior breastfeeding experiences, did that influence your decision?* | Facilitators |
| 4. I would like to ask you about your [name of person listed in question 3] and how you perceive their influence on your choice for feeding your baby. | Facilitators  or Barriers |
| 5. If any, what barriers have you encountered while breastfeeding now or in the past if you breastfed other children? | Barriers |
| 6. If any, what facilitators have you received while breastfeeding now or in the past if you breastfed other children? | Facilitators |
| 7. Can you tell me about your infant feeding methods? *Do you breastfeed? pump? Bottle? Do you use formula? solid food? or any combination?* | Infant Feeding Methods |
| 8. What, if anything, would you change about your breastfeeding experiences if you could? | N/A |
| 9. Is there anything else you would like to share or any other questions you may have? Or is there anything you may not have thought about related to the questions from the interview? | N/A |

*Note.* N/A= not applicable, * = probing questions for multigravida pregnant women

**Thank you for your time and thoughtful responses.**
