# Supplementary material for: Self-management of type 2 diabetes mellitus in pregnancy and breastfeeding experiences among women in Thailand: Study protocol
Source: PLoS One. 2023 Jun 12;18(6):e0286646. doi: 10.1371/journal.pone.0286646 (PMC10259789; doi:10.1371/journal.pone.0286646)
Supplement: S2 File — (DOCX) [file pone.0286646.s002.docx]

**SUPPLEMENT 2** EHR extraction form recording maternal health outcomes

Case ID………….

| **Maternal Health Outcomes** | **Pre Pregnancy** | **Pregnancy** | | | | **Postpartum**  **4-6 weeks** |
| --- | --- | --- | --- | --- | --- | --- |
|  |  | **First Visit**  **8-12 weeks** | **Second Visit**  **24-26 weeks** | **Third Visit**  **32 weeks** | **Forth Visit**  **36-38 weeks** |  |
| Weight (kg) |  |  |  |  |  |  |
| Height (kg) |  |  |  |  |  |  |
| BMI (kg/m^2^) |  |  |  |  |  |  |
| GWG (kg) |  |  |  |  |  |  |
| HbA1c (%) |  |  |  |  |  |  |
| FPG (mg/dL) |  |  |  |  |  |  |

*Note.* Grey box=data will not be collected*;* Thai pregnant women are required to attend at least four visits; BMI=Body Mass Index; GWG= Gestational Weight Gain; HbA1c=Glycated Hemoglobin; FPG= Fasting Plasma Glucose

| **Terms** | **Definition** |
| --- | --- |
| Body mass index (BMI) | A woman’s weight in kilograms (kg) divided by the square of height in meters (m). Asian criteria-based BMI is used as follows: <18.5 kg/m^2^ for underweight, 18.5-22.9 kg/m^2^ for normal-weight, 23.0-27.5 kg/m^2^ for overweight, and >27.5 kg/m^2^ for obese women |
| Gestational weight gain (GWG) | A woman’s total amount of weight gained during pregnancy. GWG is calculated by the difference between baseline and immediate pre-delivery body weight. The optimal weight gain should be 11.3-15.87 kg for women with BMI 18.5–24.9 kg/m^2^, 6.8–11.3 kg for women with BMI of 25–29.9 kg/m^2^, and 5–9.1 kg for women with BMI ≥ 30 kg/m^2^ |
| HbA1c level | A woman’s average blood glucose for the past two to three months. The target HbA1c level is below 6.5 percent |
| Fasting plasma glucose (FPG) level | A woman’s blood glucose after fasting at least 8 hours before the test. The target level is 80-100 mg/dl |
