# Supplementary material for: Self-management of type 2 diabetes mellitus in pregnancy and breastfeeding experiences among women in Thailand: Study protocol
Source: PLoS One. 2023 Jun 12;18(6):e0286646. doi: 10.1371/journal.pone.0286646 (PMC10259789; doi:10.1371/journal.pone.0286646)
Supplement: Attachment — Submitted filename: PONE-D-22-16726 .pdf [file pone.0286646.s003.pdf]

Manuscript Review: "Self-Management in Pregnancy and Breastfeeding in Women with Type 2 Diabetes Mellitus from Thailand: A Convergent Parallel Mixed-Methods Study Protocol".

Journal: Plos One

Journal ID: PONE-D-22-16726
Article Type: Study protocol

Item	Comments
Title	The title of the study is a bit confusing since self management in pregnancy and breastfeeding does not focus on what the study protocol is about. Suggest to change to 'Self management of diabetes care in pregnancy and breastfeeding experiences among Type 2 DM women in Bangkok, Thailand'
Abstract	The abstract is satisfactory stating the aim, the method and the significance of doing such study. Keywords are sufficient and suitable.
Introduction	The problem statement is not so clear. Suggest authors add in more info on the prevalence of DM among women in Thailand in terms of age in order to relate to the burden of the disease<br><br>Line 89-92- the factors for predictors of breastfeeding given here do not appear to be continuous with the previous matter that is concentrating on problems of breastfeeding in mothers with DM. Kindly rearrange to ensure readers can follow your arguments<br><br>Suggest to include other benefits to breastfeeding DM mothers aside from lowering glucose levels such as weight reduction etc.<br><br>Justification is stated clearly in the introduction.
Materials and method	**Study design**:<br>Appropriate study design. Variables for the quantitative study are appropriate.

	However, since the author is recruiting from one clinic from one hospital, will the study findings be generalized and represents the DM self-management and breastfeeding experience of women with DM in Thailand? Please explain.<br><br>Is the study location (hospital) where recruitment of patients will be done is a Baby friendly hospital? If yes, will it somehow impact your participants breastfeeding experience later?<br><br>In terms of recruitment of participants, what is the gestational age for the pregnant women to be recruited in this study? For an antenatal visit in the clinic, are they given information such as pamphlets, talks on breastfeeding as routine?<br><br>For exclusion criteria, if a pregnant woman develops DM eye retinopathy or other Dm complication at any stage of her pregnancy after recruitment, will they be dropped and replaced with other participants?<br><br>Line 162- PI's study office number is referring to office telephone number, is it?<br><br>Do you think the mode of delivery will have an effect on the intention and experience of breastfeeding in your study?<br><br>A question on whether the participants have had a pre-pregnancy counselling prior to getting pregnant might be useful.<br><br>**Study instruments:**<br>For the interview guide, explain who are the experts involved in developing the guide.<br><br>Are there any particular reasons to use the BSES short form rather than the longer version? Will it affect your data collection?<br><br>**Data collection**<br>1. Will there be any difference if the qualitative part of the study is done immediately after the quantitative part or later?

	2. Will there be any difference in interviewing participants face-to-face and by phone access only? My worry is that you might not be able to see the gestures, emotions, and body language, especially on the question of barriers and facilitators. Please clarify.
Table and images	Table 1- the heading should be at the top of the table
Result	NA
Discussion and conclusion	How do the data findings help in delivering patient-centred care to pregnant women with type 2 DM to breastfeed better?<br><br>Others: This article might need some proofreading in terms of English writing.
Reference	References are appropriate
