# Supplementary material for: Self-management of type 2 diabetes mellitus in pregnancy and breastfeeding experiences among women in Thailand: Study protocol
Source: PLoS One. 2023 Jun 12;18(6):e0286646. doi: 10.1371/journal.pone.0286646 (PMC10259789; doi:10.1371/journal.pone.0286646)
Supplement: Attachment — Submitted filename: PLOS ONE Manuscript PSJK review.docx [file pone.0286646.s004.docx]

**Self-Management in Pregnancy and Breastfeeding in Women with Type 2
Diabetes Mellitus from Thailand: A Convergent Parallel Mixed-Methods
Study Protocol**

**List of all authors and institutional affiliations**

**Ratchanok Phonyiam, MSN, RN (Corresponding Author)**

The University of North Carolina at Chapel Hill

School of Nursing

Campus Box 7460

Chapel Hill, North Carolina, United States 27599-7460

Email: ratch@ad.unc.edu

Telephone: 919-259-1348

Ramathibodi School of Nursing

Faculty of Medicine Ramathibodi Hospital

Mahidol University

270 Bangkok, Thailand 10400

ORCID iD: 0000-0002-2339-8889

**Marianne Baernholdt, PhD, MPH, RN, FAAN**

The University of North Carolina at Chapel Hill

School of Nursing

Campus Box 7460

Chapel Hill, North Carolina, United States 27599-7460

Email: marianne_baernholdt@unc.edu

Telephone: 919-962-8995

ORCID iD: 0000-0001-5499-7457

**Eric A. Hodges, PhD, FNP-BC, FAAN**

The University of North Carolina at Chapel Hill

School of Nursing

Campus Box 7460

Chapel Hill, North Carolina, United States 27599-7460

Email: eric.a.hodges@unc.edu

Telephone: 919-966-0534

ORCID iD: 0000-0002-4358-4398

**Funding**

This study is funded by the Alpha Alpha Chapter of Sigma Theta Tau International Research Grant and the Sigma Small Grant. The funding body was not involved in the design of the study and collection, analysis, and interpretation of data or in writing this manuscript.

**Competing interests**

The authors declare that they have no competing interests.

**Data availability**

Data sharing is not applicable to this article as no datasets were generated or analyzed. Data will be available after the completion of the study upon request to the Principal Investigator.

**Abstract**

Women with type 2 diabetes mellitus are at a higher risk of pregnancy complications. Although traditional beliefs and practices influence diabetes management and breastfeeding, recommendations integrating Thai cultural beliefs in maternal care are lacking.

This study aims to describe diabetes self-management in pregnancy and breastfeeding experiences in women with preexisting type 2 diabetes mellitus from Thailand.

A convergent parallel mixed-methods study will be conducted. Data will be collected from 20 pregnant women with preexisting type 2 diabetes mellitus in Thailand who are either primigravida or multigravida, aged 20-44 years old, speak the Thai language, and provide consent. The National Institute on Minority Health and Health Disparities Framework’s sociocultural and behavioral domains guides the research aims. Using interviews and questionnaires data will be collected two times. The first time is during pregnancy where data on a woman’s diabetes self-management, breastfeeding confidence, and breastfeeding intention will be collected. The second time is 4-6 weeks postpartum and is about her breastfeeding experiences. Maternal health outcomes will be collected from electronic health records at each time point. Qualitative data will be analyzed using directed content analysis. Quantitative data will be analyzed using descriptive statistics. Data sources will be triangulated with relative convergence in the results. This proposed study is significant because the findings will have a potential impact on improving two generations' health across a lifespan. Health care providers will apply the findings to deliver holistic care to women with type 2 diabetes mellitus.

**Keywords**

Self-management, Pregnancy, Postpartum, Breastfeeding, Women, Type 2 diabetes mellitus

**Introduction**

Globally, the diabetes prevalence is 10.5% (537 million adults) in 2021 [1]. Thailand is among the countries with a higher prevalence rate in women (10.8%) than men (8.9%) [2]. The number of women with type 2 diabetes mellitus (T2DM) who become pregnant has increased [1]. Pregnancy with T2DM is associated with a two-fold increased risk of pre-eclampsia [3] and a three-fold increased risk of both miscarriage and perinatal mortality [4]. Exposure to maternal complications contributes to higher odds of fetal macrosomia as a birth weight of more than 4,000 grams (Odd Ratios [OR] 5.12, 95% Confidence Interval [CI] 4.72−5.54), respiratory distress syndrome (OR 3.07, 95% CI 2.75−3.43), and neonatal death (OR 2.49, 95% CI 1.40−4.44) [5]. These adverse outcomes can be minimized with proper diabetes self-management during pregnancy.

Thai traditional beliefs and practices influence diabetes management behaviors, serving as either a barrier or facilitator [6, 7]. For example, tradition dictates that pregnant women must take many precautions, including avoiding certain foods and rigorous activity to ensure their unborn child's safety and well-being [8]. Further, typical Thai meals are served together with rice, so the restriction of carbohydrates in T2DM creates a unique challenge. Balancing dietary and physical activity to achieve optimal glycemic control is a challenge [9]. An appreciation of the cultural influences on how Thai persons with diabetes view their condition facilitated self-management [7], which in turn, may improve maternal and fetal health.

In addition to challenges for maternal health during pregnancy for women with T2DM, mothers with T2DM commonly experienced difficulty with milk supply, delayed lactation [10] and have worries about hypoglycemia episodes while breastfeeding [11]. Mothers with T2DM are less likely to establish exclusive breastfeeding at hospital discharge (OR 0.4; 95% CI 0.2, 0.8; *p* = 0.006), compared to women without T2DM [12]. Further, the number of feedings in the first 24 hours was an independent positive predictor of breastfeeding four months postpartum (OR 1.158; 95% CI 1.021–1.314, *p* = 0.022), while maternal body mass index was a negative predictor (OR 0.848; 95% CI: 0.779–0.922, *p* < 0.001) [11].

Breastfeeding benefits have been widely recognized for mothers with diabetes. Early breastfeeding initiation optimizes maternal glucose regulation. A systematic review found that mothers who breastfeed their infants have either reduced or comparable insulin requirements than those who bottle-fed [13]. The mechanisms are threefold. First,suckling directly influence maternal blood glucose levels through glucose uptake into the breast or indirectly via lactogenic hormones such as oxytocin and prolactin [13]. Second, at each breastfeeding episode, oxytocin expresses into skeletal muscle via the oxytocin receptor and stimulates insulin secretion [14]. Finally, breastfeeding women had significantly higher prolactin levels than women who did not (*p* < 0.001) as Prolactin acts directly on pancreatic beta-cells and increases insulin concentration [13]. Prolactin was negatively correlated with HbA1c and 2-hour serum C-peptide (*r* = −0.564, *p* = 0.005, *r* = −0.539, *p* = 0.008, respectively) [15].

The World Health Assembly has endorsed a global target for increasing the exclusive breastfeeding (EBF) rate in the first six months up to at least 70% by 2030 [16]. In a 2019 survey, only 14% of all Thai infants aged 0-6 months were exclusively breastfed [17]. The six-month EBF rate in Thailand is still far from optimal. Little is known about how diabetes affects a woman’s confidence and intention to breastfeed her infant and her breastfeeding experiences. Gaining a better understanding of Thai women’s diabetes management experiences during pregnancy and breastfeeding practices postpartum will inform evidence‐based clinical approaches to enhance the health of women with T2DM and their children in Thailand.

**Materials and Methods**

**Aims**

This study is guided by the social and cultural domains in the National Institute on Minority Health and Health Disparities (NIMHD) Framework [18]. This framework provides an understanding of sociocultural and behavioral domains of influence at individual, interpersonal, and community levels. The specific aims are 1) to describe attitudes and confidence toward diabetes self-management in Thai pregnant women with preexisting T2DM, 2) to describe the barriers and facilitators of diabetes self-management in pregnancy, 3) to describe breastfeeding confidence and intention in pregnancy, and 4) to describe breastfeeding barriers and facilitators in women 4-6 weeks postpartum.

**Study design**
This study uses a convergent parallel mixed-methods design [19]. Qualitative and quantitative data will be collected concurrently [19]. The rationale for this mixed methods design is to bring together the results of qualitative and quantitative data to obtain a more comprehensive understanding of women’s diabetes self-management in pregnancy and breastfeeding experiences [19] In this study, qualitative elements are the primary methods used to answer the research questions; at the same time, supplementary data from quantitative elements will deepen the findings [19]. The qualitative data will be derived from a qualitative descriptive approach [20, 21]. The qualitative description is an appropriate qualitative approach to answer our research questions because we will discover the who, what, and where of diabetes management and breastfeeding experiences by gaining insights from women with T2DM. The quantitative data will employ a descriptive design using self-reported questionnaires and physiological measures from the Electronic Health Records (EHRs).

**Participant eligibility and recruitment**

This study will recruit pregnant women with T2DM receiving antenatal care from one hospital clinic located in Bangkok, Thailand. Our research team will identify potential participants from EHRs. Recruitment will be achieved through face-to-face outreach in a private room at the clinic or over the phone.

A purposive sample will be used to enroll participants. Women will be eligible to join if they are experienced with T2DM self-management during pregnancy [22]. The inclusion criteria will be 1) pregnant women with preexisting T2DM, defined as either pregestational type 2 or overt diabetes mellitus requiring medical treatment such as oral agents or insulin injection, 2) either primigravida or multigravida, 3) reproductive age (20-44 years old), 4) able to speak the Thai language, and 5) able to provide consent. Participants will be excluded if they have any significant complications such as blindness or life-threatening illnesses such as myocardial infarction. Our goal is to seek a maximally diverse group of pregnant women with T2DM using maximum variation sampling [22] to capture various experiences that emerged from women’s different baseline of HbA1c level, gravidity, and trimester. These interest criteria were derived from the literature focused on pregnant women who will have various experiences on diabetes self-management in pregnancy [23, 24].

**Ethical considerations**

Ethical approval has been obtained from the University of North Carolina at Chapel Hill (IRB: 21-1477) and the Mahidol University in Thailand (IRB number 3428).

Before the potential participants decide to participate in this study, either the principal investigator (PI) or a trained research assistant will explain the objectives, data collection procedures, benefits, and impact of this study. Participants will be informed in the written consent to have data from their medical records used in this study. Participants' rights to participate or withdraw from the study without any effect on care will be emphasized. The PI will give the women a chance to ask questions before deciding to participate in this study. Written informed consent will be obtained during participants’ enrolment. All participants will receive a signed copy of the consent form, which includes the PI’s study office number. They will be encouraged to call with any questions or concerns and the phone numbers for the offices of human subject protection for any questions they may have regarding their rights as study participants. Participants who are unable to give informed consent will also be excluded from the study.

**Sample size**

As this study is descriptive rather than comparative in nature, statistical significance does not drive the determination of target sample size. Alternatively, the sample size will initially focus on conducting approximately 20 semi-structured interviews, but the final sample size will be determined by data saturation [22].

**Instruments**

**Interview guide**

Our research team developed an interview guide in collaboration with experts in women’s health and diabetes self-management. The interview guide lists open-ended and follow-up questions. Before study implementation, the interview guide will be pilot tested with two pregnant women with T2DM in Thailand. The interviewer will ask for feedback on the clarity of interview questions and probes, and the interview guide will be revised as needed. Each woman will be asked the same questions in the same order (Supplement 1).

**Questionnaires**

For Time 1, each woman will response to three questionnaires as follows.

A demographic questionnaire includes a mother’s age, marital status, educational level, monthly household income, employment status, duration of diabetes, trimester of pregnancy, comorbid conditions, and current medications.

We will use the Thai version of the 14-item Breastfeeding Self-Efficacy Scale-Short Form (BSES-SF; Cronbach’s alpha = 0.84) to measure a woman’s confidence to breastfeed her infant [25]. The BSES-SF used the 5-point Likert-type scale where 1 = not at all confident and 5 = very confident. Item ratings will be summed to produce a total score from 14 to 70, with higher scores indicating higher confidence.

We will use the Thai version of the 5-item Infant Feeding Intentions Scale (T-IFI; Cronbach’s alpha = 0.86) to measure maternal intention to breastfeed her infant [26]. The original Cronbach’s alpha was 0.90 [27]. The T-IFI used the 5-point Likert-type scale, individually scored from 0 to 4. Total IFI score is calculated by averaging the score for the first two items and summing this average with the scores for items 3–5. The possible score ranges from 0 to 16, with 0 representing a very strong intention not to breastfeed and 16 representing a very strong intention to fully breastfeed as the sole source of milk until six months of age.

**Physiological measures**

Upon enrollment into the study, a woman’s gestation will be established, and biomarker data will be collected retrospectively and prospectively according to the schedule detailed in Supplement 2. Biomarker data include maternal body mass index (BMI), gestation weight gain (GWG), HbA1c, and fasting plasma glucose (FPG). Thai women are advised and encouraged to attend the four-visit focused ANC (FANC) model at 8-12 weeks, 24-26 weeks, 32 weeks, and 36-38 weeks of gestation. We will also collect the HbA1c level; a woman’s average blood glucose for the past two to three months. Additionally, HbA1c level from prepregnancy and at 24-26 weeks ANC visit will be collected.

We will extract maternal FPG levels measured at 4-6 weeks postpartum from the EHR. FPG is a woman’s blood glucose level after fasting at least eight hours before the test. The target level is 80-100 mg/dl [9].

**Data collections**

Before a woman decides to participate in this study, we will explain the objectives, data collection procedures, benefits, and the study’s impact. Participants' rights to participate or withdraw from the study at any time without effect on care will be emphasized. We will give the women a chance to ask questions before deciding whether to participate. Written consent will be obtained from all participants.

**Time 1 data collection**

At time 1 the pregnant women will complete questionnaires and engage in an interview about diabetes self-management and breastfeeding intention. Data collection will take 1- 1½ hours. Participants will first be asked to fill out the demographic, BSES-SF, and T-IFI questionnaires which will take approximately 15 minutes. The participants will then participate in an interview at that time or later either by phone, Zoom call with no video on/phone access only, or in-person according to their preference. In-person recorded interviews will be conducted in a private room in the antenatal clinic, and each interview will take approximately 60 minutes. The interviewer will follow an interview guide. The interviewer will also write field notes about the environment and participant observation during the interview. For virtual interviews, phone or a Zoom call with no video on/phone access only will be used. For phone interviews the Voice Memos app will be used to audio-record the interview. The Zoom or phone recordings will not include names, telephone numbers, medical record numbers, or other identifiable items. The quality of the recording will be reviewed after each interview. The interviewer will then complete reflective notes to summarize the interview's overall quality and factors contributing to interview quality for every interview regardless of interview means.

**Contacts between time 1 and time 2**

After time 1, we will ask for a woman’s and a couple of her family members’ phone numbers and permission to call family members if we cannot contact the participant. To maximize retention, we will do monthly check-in calls according to a woman’s preferred communication mode (a phone call, or a text message) until the time 2 data collection.

**Time 2 data collection**

Time 2 data collection is at 4-6 weeks postpartum and is an open-ended interview about breastfeeding experiences and infant feeding methods such as exclusive breastfeeding (e.g., direct breastfeeding and pumping and bottle feeding) and non-exclusive breastfeeding (e.g., formula feeding). The second interview will be conducted by phone. Each interview will take approximately 60 minutes. A mother’s fasting plasma glucose will be measured at the 4-6 weeks postpartum visit.

Participants will receive a $10 gift card after each data collection section for a total of $20. The schedule of data collection procedures and forms is summarized in Table 1.

**TABLE 1** Schedule of procedures and form

| **Procedures/Forms** | **Data Source** | **Recruitment** | **T1** | **FANC*** | **T2** | **Alpha** |
| --- | --- | --- | --- | --- | --- | --- |
| Eligibility screening | PI, RA, RNs | X |  |  |  |  |
| Enrollment | PI, RA |  | X |  |  |  |
| Inform consent | Participants |  | X |  |  |  |
| Demographic | Participants |  | X |  |  |  |
| **Aim 1** |  |  |  |  |  |  |
| First interviews | Participants |  | X |  |  |  |
| HbA1c (prepregnancy) | Chart review |  | X |  |  |  |
| **Aim 2** |  |  |  |  |  |  |
| First interviews | Participants |  | X |  |  |  |
| HbA1c (pregnancy) | Chart review |  | X |  |  |  |
| Body mass index | Chart review |  | X | XXX |  |  |
| Gestational weight gain | Chart review |  | X | XXX |  |  |
| **Aim 3** |  |  |  |  |  |  |
| First interviews | Participants |  | X |  |  |  |
| BSES-SF | Participants |  | X |  |  | .84 |
| T-IFI | Participants |  | X |  |  | .90 |
| **Aim 4** |  |  |  |  |  |  |
| Second interviews | Participants |  |  |  | X |  |
| Fasting plasma glucose | Chart review |  |  |  | X |  |
| Infant feeding methods | Participants |  |  |  | X |  |
| **Rigor and trustworthiness** | | | | | | |
| Field notes | PI |  | X |  | X |  |
| Member checking | Participants |  | X |  |  |  |

*Note.* T1=Pregnancy; FANC=Four-visit focused antenatal care; T2=One-month postpartum; PI=Principal Investigator; RNs=Registered Nurses; T-IFI=Thai Infant Feeding Intention Scale; BSES-SF=Breastfeeding Self-Efficacy Scale-Short Form; HbA1c=Glycated Hemoglobin
*Timing of FANC may vary based on a woman’s visit schedule and enrollment, but body mass index and gestational weight gain must be collected by chart review at least once.

**Data management**

For qualitative data, we will transcribe the audiotape verbatim in the Thai language. To ensure the transcription quality, the interviewer will review the Thai audio recordings along with the electronic transcript. Additional information (e.g., silences, pauses, and laughing) will be noted in each transcribed interview. All identifying information will be removed, and it will then be saved as a Microsoft Word file. Second, the transcribed interview will be sent to a bilingual professional translation service to translate into English.

By using a Research Electronic Data Capture (REDCap) mobile to collect quantitative data, all data will be automatically entered into ta REDCap database. We will track participants using case identification (ID) numbers. We will store the participants' names, telephone numbers, and EHR IDs in a separate file. We will store interview data (audio files) and transcribed interviews on a secure server. We will create an audit trail with a transparent description of our study, with field notes, data collection processes, and data analysis decisions. Assurances of protection of individual participant privacy and confidentiality will be given. All data will be kept in a locked file cabinet or computer behind a locked door. Data results will be reported in aggregate form only.

**Data analysis**

**Qualitative data**

Qualitative data analysis will occur concurrently with data collection as an iterative process. First, English transcripts will be analyzed using Atlas.ti version 9, which will facilitate data organization and joint analysis of the text. Data analysis will be based on directed content analysis [28]. The NIMHD framework’s domains (sociocultural environment and behavioral influences at individual, interpersonal, and community levels) will be used to guide initial coding. Two coders will code interview transcripts independently. We will identify recurring patterns of participants’ words within the data based on relevance to the predetermined domains of inquiry. Data that cannot be coded as one of the NIMHD framework’s domains will be identified and analyzed later to determine if they represent a new category or a subcategory of an existing code [28]. Descriptive coding will be used to assign a single code to a chunk of data (2-3 lines) with stand-alone meaning. The coders will analyze the codes and investigate how different codes can be combined to form an overarching theme, with the visual representation using a mind map from Atlas.ti.

The analysis will involve a constant moving back and forth between the entire dataset and individual transcript coding. The coders will work systematically through the dataset, give equal attention to each transcript, and identify interesting aspects that could form the basis of repeated patterns across the dataset. The coders will then describe a clear definition and name for each theme by identifying each theme's essence and determining each theme's aspect that could capture diabetes self-management and breastfeeding experiences. A table will be developed to present themes, subthemes, and quotations.

Throughout data analysis, the coders will write memos, writing down ideas and potential coding schemes. A record of all decisions made in the data analysis process will be recorded in the audit trail [29]. The two coders will have weekly meetings to assess the interview process, review the transcripts, and evaluate amount of new information to confirm when data saturation is achieved. Discrepancies will be critically examined and compared against the original transcripts and field notes until a consensus is reached. Discrepancies will be also solved by a third researcher who has the expertise in qualitative method.
 **Quantitative data**
 Data from the questionnaires and EHR extractions will be entered and analyzed using IBM SPSS version 25.0. Data will be screened for inconsistent or abnormal values. Continuous measures will be assessed for normality and outliers. Analyses include calculation of means, standard deviations, medians, and ranges for continuous variables and frequencies and percentages for categorical variables.

**Triangulation of qualitative and quantitative data sources**

Triangulation is desirable in mixed methods research because it serves as validation and confirmation of the phenomena being studied [30]. To conduct triangulation, qualitative and quantitative analyses will be conducted separately. The results will then be compared and contrasted to identify data convergence and divergence (Fig 1) [19].

The results will be presented in a side-by-side joint display (Table 2). Qualitative themes will be shown in column 1, while column 2 represents the quantitative findings (e.g., frequencies, means, and standard deviations). By using a side-by-side comparison, our research team will analyze and triangulate how the findings represent convergence or divergence between the two data sources [19].

**TABLE 2** Examples for a joint display

| **Convergent Parallel Mixed-Methods** | | **Quantitative results**  (e.g., frequencies, means, and standard deviations) | | | | | |
| --- | --- | --- | --- | --- | --- | --- | --- |
|  |  | HbA1c* | BSES-SF | T-IFI | GWG | FPG | EBF |
| **Qualitative themes** | Attitude toward diabetes | - or + |  |  |  |  |  |
|  | Confidence toward diabetes | - or + |  |  |  |  |  |
|  | Diabetes self-management barriers | - or + |  |  |  |  |  |
|  | Diabetes self-management facilitators | - or + |  |  |  |  |  |
|  | Breastfeeding confidence |  | **+** |  | - or + |  | - or + |
|  | Breastfeeding intention |  |  | **+** | - or + |  | - or + |
|  | Breastfeeding barriers |  |  |  |  | - or + | - |
|  | Breastfeeding facilitators |  |  |  |  | - or + | **+** |

*Note:* Convergence noted by plus sign (+). Divergence noted by negative sign (-).
HbA1c=Glycated Hemoglobin; BSES-SF=Breastfeeding Self-Efficacy Scale-Short Form; T-IFI: Thai Infant Feeding Intentions Scale; GWG=Gestational Weight Gain; FPG=Fasting Plasma Glucose; EBF=Exclusive Breastfeeding (Direct Breastfeeding and Pumping and Bottle Feeding)

*Timing of HbA1c levels from prepregnancy and pregnancy may vary based on a woman’s visit schedule and data availability.

**DISCUSSION**

The study described in this protocol will provide a better understanding of Thai women’s diabetes self-management during pregnancy and breastfeeding practices in their postpartum period. The strength of using a mixed-method approach is to adequately capture multiple dimensions and increase the depth of understanding of women’s experiences from both qualitative and quantitative results.

We recognize that our study protocol has limitations. First, the structure of the interview guide may influence the responses of the interviewee because of predetermined interview questions. Therefore, we included open-ended questions that will be pilot tested. Second, the process of extracting maternal health outcomes from the EHR may threaten the overall reliability of findings as the research team may extract data inaccurately. Therefore, we have developed a study protocol and an extraction form to recode EHR data. The research team members will be trained to conduct the EHR review.

In summary, this study will be used as a preliminary guide to develop a tailored approach to address barriers, strengthen facilitators, and enhance women’s health. This study will provide information for health care providers on how deliver patient-centered care. Our findings may be used as a protocol example when new guidelines are tailored for Asian regions and culturally tailored to different cultural beliefs. The findings will eventually inform policy in delivering a more integrative care model to improve maternal and infant health in Thailand.

**Author contributions**

RP, MB, EAH: Made substantial contributions to conception and design. RP, MB, EAH: Involved in drafting the manuscript and revising it critically for essential intellectual content. RP, MB, EAH: Given final approval of the version to be published. RP, MB, EAH: Agreed to be accountable for all aspects of the work in ensuring that questions related to the accuracy or integrity of any part of the work are appropriately investigated, discussed, and resolved.

**Acknowledgments**

The authors would like to thank Diane C. Berry, PhD, ANP-BC, FAANP, FAAN who made significant contributions to conception and design, involved in drafting the manuscript, and provided useful feedback and discussions that helped shape this manuscript.

**References**

1.	International Diabetes Federation. IDF Diabetes Atlas 2021 2021 [Available from: <https://diabetesatlas.org/atlas/tenth-edition/>.

2.	Aekplakorn W, Chariyalertsak S, Kessomboon P, Assanangkornchai S, Taneepanichskul S, Putwatana P. Prevalence of diabetes and relationship with socioeconomic status in the Thai population: National Health Examination Survey, 2004–2014. Journal of Diabetes Research. 2018;2018:1-8.

3.	Persson M, Cnattingius S, Wikström A, Johansson S. Maternal overweight and obesity and risk of pre-eclampsia in women with type 1 diabetes or type 2 diabetes. Diabetologia. 2016;59(10):2099-105.

4.	Mukerji G, Bacon S, Feig DS. Gestational diabetes and type 2 diabetes during pregnancy. Maternal-Fetal and Neonatal Endocrinology: Elsevier; 2020. p. 371-88.

5.	Battarbee AN, Venkatesh KK, Aliaga S, Boggess KA. The association of pregestational and gestational diabetes with severe neonatal morbidity and mortality. J Perinatol. 2020;40(2):232-9.

6.	Auemaneekul N, Chandanasotthi P, Rawiworrakul T, Sowattanangoon N, Boonsatean W. Community partnership, context-based intervention and diabetes control in Thailand. Journal of Public Health. 2016;46(3):236-47.

7.	Sowattanangoon N, Kotchabhakdi N, Petrie KJ. The influence of Thai culture on diabetes perceptions and management. Diabetes Research and Clinical Practice. 2009;84(3):245-51.

8.	Liamputtong P, Kitisriworapan S. Authoritative knowledge, folk knowledge, and antenatal care in contemporary Northern Thailand. In: Liamputtong P, editor. Contemporary socio-cultural and political perspectives in Thailand. Springer: Dordrecht; 2014.

9.	American Diabetes Association. 14. Management of diabetes in pregnancy: Standards of medical care in diabetes—2021. Diabetes Care. 2021;44(Suppl1):S200-S10.

10.	De Bortoli J, Amir LH. Is onset of lactation delayed in women with diabetes in pregnancy? A systematic review. Diabet Med. 2016;33(1):17-24.

11.	Herskin CW, Stage E, Barfred C, Emmersen P, Ladefoged Nichum V, Damm P, et al. Low prevalence of long-term breastfeeding among women with type 2 diabetes. The Journal of Maternal-Fetal & Neonatal Medicine. 2016;29(15):2512-7.

12.	Longmore DK, Barr ELM, Barzi F, Lee IL, Kirkwood M, Connors C, et al. Social and economic factors, maternal behaviours in pregnancy and neonatal adiposity in the PANDORA cohort. Diabetes Research and Clinical Practice. 2020;161:108028.

13.	Achong N, Duncan EL, McIntyre HD, Callaway L. The physiological and glycaemic changes in breastfeeding women with type 1 diabetes mellitus. Diabetes Research and Clinical Practice 2018;135:93-101.

14.	Uvnäs Moberg K, Ekström-Bergström A, Buckley S, Massarotti C, Pajalic Z, Luegmair K, et al. Maternal plasma levels of oxytocin during breastfeeding-A systematic review. PLoS One. 2020;15(8):e0235806.

15.	Ozisik H, Suner A, Cetinkalp S. Prolactin effect on blood glucose and insülin in breastfeeding women. Diabetol Metab Syndr. 2019;13(3):1765-7.

16.	WHO/UNICEF. The extension of the 2025 Maternal, Infant and Young Child nutrition targets to 2030 2021 [Available from: <https://www.who.int/nutrition/global-target-2025/discussion-paper-extension-targets-2030.pdf?ua=1>.

17.	World Health Organization. Exclusive breastfeeding under 6 months Data by country 2019 [Available from: <https://apps.who.int/gho/data/view.main.NUT1730>.

18.	Alvidrez J, Castille D, Laude-Sharp M, Rosario A, Tabor D. The National Institute on Minority Health and Health Disparities Research Framework. Am J Public Health. 2019;109(S1):S16-s20.

19.	Creswell JW, Creswell JD. Research design qualitative, quantitative, and mixed methods approaches (5th edition). Thousand Oaks: SAGE Publications, Inc.; 2018.

20.	Sandelowski M. Whatever happened to qualitative description? Res Nurs Health. 2000;23:334-40.

21.	Sandelowski M. What's in a name? Qualitative description revisited. Res Nurs Health. 2010;33(1):77-84.

22.	Patton M. Qualitative research and evaluation methods: Integrating theory and practice (4th ed.). Newbury Park, CA Sage Publications; 2014.

23.	Collier SA, Mulholland C, Williams J, Mersereau P, Turay K, Prue C. A qualitative study of perceived barriers to management of diabetes among women with a history of diabetes during pregnancy. J Womens Health. 2011;20(9):1333-9.

24.	Lavender T, Platt MJ, Tsekiri E, Casson I, Byrom S, Baker L, et al. Women's perceptions of being pregnant and having pregestational diabetes. Midwifery. 2010;26(6):589-95.

25.	Thussanasupap B. The effects of systematic instructional program on breastfeeding self-efficacy, nipple pain, nipple skin changes and incision pain of cesarean mothers: Mahidol University; 2006.

26.	Phonyiam R, D. K, A.E.L. P. Cross-cultural adaptation and validation of the Infant Feeding Intentions Scale for Thai pregnant women. 2022.

27.	Nommsen-Rivers LA, Dewey KG. Development and validation of the infant feeding intentions scale. Maternal and Child Health Journal. 2009;13(3):334-42.

28.	Hsieh HF, Shannon SE. Three approaches to qualitative content analysis. Qual Health Res. 2005;15(9):1277-88.

29.	Lincoln YS, Guba EG. Naturalistic inquiry. Newbury Park, CA: Sage Publications; 1985.

30.	Denzin NK. The research act (3rd ed.). Englewood Cliffs, NJ: Prentice Hall; 1989.
