# Supplementary material for: Self-management of type 2 diabetes mellitus in pregnancy and breastfeeding experiences among women in Thailand: Study protocol
Source: PLoS One. 2023 Jun 12;18(6):e0286646. doi: 10.1371/journal.pone.0286646 (PMC10259789; doi:10.1371/journal.pone.0286646)
Supplement: Attachment — Submitted filename: PONE Response to Reviewers (1-23-2023).docx [file pone.0286646.s005.docx]

**PONE-D-22-16726**

**Self-management of type 2 diabetes mellitus in pregnancy and breastfeeding experiences among women in Thailand: Study protocol**

Dear Reviewers,

We appreciate the opportunity to address your comments and to revise this manuscript. Below please find item-by-item responses to those comments, which are included verbatim. We have indicated all revisions by using text changes, and all page and line numbers refer to locations in the revised manuscript (PONE-D-22-16726.R1). We believe the manuscript has been considerably strengthened with these revisions.

| **Reviewer 1 Comment** | **Author Response** |
| --- | --- |
| Reviewer #1: In general, this study seems feasible to be done. However, there are some comments that is needed to be clarified by the author. Kindly see the attached comments for full review. | Thank you for your thoughtful comments. We have carefully considered all the issues brought to our attention and made necessary changes point by point. Please see the revised submission in the attached document. |
| The title of the study is a bit confusing since self management in pregnancy and breastfeeding does not focus on what the study protocol is about. Suggest to change to ‘Self management of diabetes care in pregnancy and breastfeeding experiences among Type 2 DM women in Bangkok, Thailand’ | We revised our manuscript title to  “Self-management of type 2 diabetes mellitus in pregnancy and breastfeeding experiences among women in Thailand: Study protocol”  Please see Lines 1-2. |
| The abstract is satisfactory stating the aim, the method and the significance of doing such study. Keywords are sufficient and suitable. | Thank you so much. |
| The problem statement is not so clear. Suggest authors add in more info on the prevalence of DM among women in Thailand in terms of age in order to relate to the burden of the disease | We provided the statistics from a recent Thai national survey data as suggested. Please see Lines 111-113. |
| Line 89-92- the factors for predictors of breastfeeding given here do not appear to be continuous with the previous matter that is concentrating on problems of breastfeeding in mothers with DM. Kindly rearrange to ensure readers can follow your arguments | We removed the predictors (e.g., numbers of feeding and maternal body mass index) as suggested. Please see Lines 162-164. |
| Suggest to include other benefits to breastfeeding DM mothers aside from lowering glucose levels such as weight reduction etc. | We added breastfeeding benefits including reducing triglycerides, increasing HDL cholesterol, and reducing postpartum maternal weight.  Citations were added accordingly. Please see Lines 210-212. |
| Justification is stated clearly in the introduction. | Thank you so much. |
| **Study design:**  Appropriate study design. Variables for the quantitative study are appropriate. | Thank you so much. |
| However, since the author is recruiting from one clinic from one hospital, will the study findings be generalized and represents the DM self-management and breastfeeding experience of women with DM in Thailand? Please explain. | The study’s findings will not be able to be generalized because of the small sample size and a single data collection site. Rather, our goal is transferability since our findings can be applied and transferred to other contexts and samples. We added our explanations and a citation in the manuscript text to deepen our response. Please see Lines 576-579. |
| Is the study location (hospital) where recruitment of patients will be done is a Baby friendly hospital? If yes, will it somehow impact your participants breastfeeding experience later? | Yes. Most hospitals in Thailand are baby-friendly hospitals (Lines 216-217). We think that will have a positive impact on breastfeeding experiences among participants.  Since this is a Baby-Friendly Hospital, healthcare clinicians may be more interested in improving and sustaining breastfeeding  Please see Lines 262-264. |
| In terms of recruitment of participants, what is the gestational age for the pregnant women to be recruited in this study? For an antenatal visit in the clinic, are they given information such as pamphlets, talks on breastfeeding as routine? | Gestational age is not one of our eligibility criteria. We are not limiting gestational age because our goal is to seek a maximally diverse group of pregnant women with type 2 diabetes mellites to capture experiences that emerged in adapting to gestational age across trimesters. Please see Lines 307-310.  Pregnant women receive the maternal and child health book or “Pink Book” when they access antenatal clinic during their first visit. The Pink Book provides health information and health services from pre-conception until the child is 5 years old. The services include counseling on breastfeeding and nutrition. Please see Lines 314-317. |
| For exclusion criteria, if a pregnant woman develops DM eye retinopathy or other Dm complication at any stage of her pregnancy after recruitment, will they be dropped and replaced with other participants? | After enrollment, if a woman developed diabetes complications such as retinopathy, she will not be dropped unless she wishes to withdraw from our study. If a woman withdraws, we will recruit another participant. Please see Lines 305-307. |
| Line 162- PI’s study office number is referring to office telephone number, is it? | Thank you for pointing this out.  Yes. We referred to the PI’s office telephone number. However, we removed this statement based on reviewer 2's comment.  Please see Lines 348-349. |
| Do you the mode of delivery will have  an effect on the intention and experience of breastfeeding in your study? | Yes. We do think this might be the case. However, the mode of delivery may not affect a woman’s intention to breastfeed as she may not know if she will deliver a baby vaginally or undergo a cesarean section. We agree and are aware that the mode of delivery will have an impact on breastfeeding experiences **after** childbirth. For example, mothers who had cesarean section may have delayed lactogenesis compared to mothers who had normal labor. Delayed lactogenesis may result in a reduced frequency of breastfeeding in early postpartum.  Thus, our research team anticipates seeing differences in breastfeeding intention and experiences from our participants based on their mode of delivery. |
| A question on whether the participants have had a pre-pregnancy counselling prior to getting pregnant might be useful. | We agree with your comment.  We added a statement related to our interview questions. Please see Line 362. |
| **Study instruments:**  For the interview guide, explain who are the experts involved in developing the guide. | We added more details on how our interview guide was developed as well as the translation process. Please see Lines 361-369. |
| Are there any particular reasons to use the BSES short form rather than the longer version? Will it affect your data collection? | The Breastfeeding Self-Efficacy Scale-Short Form (BSES-SF; Cronbach’s alpha = 0.84) is as good as the long version and culturally appropriate for Thai women. The rationale for using the short version is to reduce participants’ burden. We added our justification. Please see Lines 385-387. |
| **Data collection:**  1. Will there be any difference if the qualitative part of the study is done immediately after the quantitative part or later? | Yes. There is a possibility that qualitative results from the interview would be different if participants engage in an interview immediately or later after responding to our questionnaires. The interview appointment is based on a woman’s availability for a 60-minute interview. Please see Lines 424-425.  The interviewer will write field notes about the interview date and time. We will use each field note in our qualitative data analysis to articulate any findings’ differences between immediately after the quantitative part or later. Please see Lines 435-436. |
| 2. Will there be any difference in interviewing participants face-to-face and by phone access only? My worry is that you might not be able to see the gestures, emotions, and body language, especially on the question of barriers and facilitators. Please clarify. | We agree that having a phone interview will not allow the interviewer to see and observe a woman’s gestures, facial expressions, and body language.  The interviewer will write field notes about the modes of interview. We will incorporate our field notes into our qualitative data analysis to articulate any differences according to interview mode. Lines 428-429 and 435. |
| Table 1- the heading should be at the top of the table | We made changes as suggested. |
| How do the data findings help in delivering patient-centred care to pregnant women with type 2 DM to breastfeed better? | We added the implications of our findings. Please see a revision Lines 580-585. |
| Others: This article might need some proofreading in terms of English writing | We appreciate your comment. |
| References are appropriate | Thank you so much |

| **Reviewer 2 Comment** | **Author Response** |
| --- | --- |
| The term 'breastfeeding' is rather broad. Suggest to write 'breastfeeding experiences' in parallel with the study objectives. | We revised our manuscript title. Please see Lines 1-2. |
| **Abstract:**  This study has two Major outcomes/ dependant variables which does not seem to be related, as these outcomes are rather major and encompasses many aspects on its own. Suggest to focus on one major outcome, i.e breastfeeding. | Thank you so much for pointing this out. This study has four aims as discussed in the manuscript text. We revised this statement to reflect the overarching purpose of our study. Please see Lines 54-55. |
| "Data will be collected from 20 pregnant women with preexisting type 2 diabetes mellitus in Thailand who are either primigravida or multigravida, aged 20-44 years old, speak the Thai language, and provide consent."- Provide consent- can be stated in methods, not in abstract. | This is one of our inclusion criteria. Thus, after careful consideration, we have not removed this statement. Please see Lines 58-59. |
| "Using interviews and questionnaires data will be collected two times. The first time is during pregnancy where data on a woman’s diabetes self-management, breastfeeding confidence, and breastfeeding intention will be collected. The second time is 4-6 weeks postpartum and is about her breastfeeding experiences. Maternal health outcomes"- grammatical error. | Thank you very much for pointing this out. We made editorial changes. Please see Lines 60-61. |
| "The first time is during pregnancy where data on a woman’s diabetes self-management, breastfeeding confidence, and breastfeeding intention will be collected. The second time is 4-6 weeks postpartum and is about her breastfeeding experiences.It is not clear on how data collection is going to be conducted during these two time-points."- Suggest to briefly elaborate which outcome will be assessed using quantitative and qualitative data respectively. | We revised how we will collect data at each time point. Please see Lines 61-64. |
| What ‘health outcomes’ in particular? Suggest to state briefly in abstract. | We clarified what health outcomes we collected. Please see Lines 64-66. |
| "This proposed study is significant because the findings will have a potential impact on improving two generations' health across a lifespan. Health care providers will apply the findings to deliver holistic care to women with type 2 diabetes mellitus."-There is lack of strong justification provided by author. Suggest to rephrase to highlight the unique contribution of this study. | We made changes as suggested.  Please see Lines 68-70. |
| **Introduction** The introduction should not be presented with detailed statistics as such. Suffice to briefly elaborate the complications with appropriate citations. | We removed the statistics as suggested. Please see Lines 116-118. |
| “These adverse outcomes can be minimized with proper diabetes self-management during pregnancy."- citation needed. | Citation was added (Line 119). |
| "An appreciation of the cultural influences on how Thai persons with diabetes view their condition facilitated self-management [7], which in turn, may improve maternal and fetal health."- I don’t quite understand this statement. | We edited the sentence (Lines 125-127). |
| "Mothers with T2DM are less likely to establish exclusive breastfeeding at hospital discharge (OR 0.4; 95% CI 0.2, 0.8; p = 0.006), compared to women without T2DM [12]."- Refer to my earlier comment on presentation of detailed statistics in introduction which is unnecessary. | We removed the statistics as suggested. Please see Lines 160-162. |
| "Further, the number of feedings in the first 24 hours was an independent positive predictor of breastfeeding four months postpartum (OR 1.158; 95% CI 1.021–1.314, p = 0.022), while maternal body mass index was a negative predictor (OR 0.848; 95% CI: 0.779–0.922, p < 0.001) [11]."- This statement seemed to be disjointed. Suggest to add on/ elaborate on other breastfeeding challenges that mothers with T2DM may face (rather than highlighting on the number of feeds and BMI which seemed to be irrelevant. | You have raised an important point. We added other breastfeeding challenges such as infant. Citations were added accordingly. We also removed the statement about the number of feedings and maternal body mass index. Please see Lines 164-169. |
| "The six-month EBF rate in Thailand is still far from optimal. Little is known about how diabetes affects a woman’s confidence and intention to breastfeed her infant and her breastfeeding experiences. Gaining a better understanding of Thai women’s diabetes management experiences during pregnancy and breastfeeding practices postpartum will inform evidence‐based clinical approaches to enhance the health of women with T2DM and their children in Thailand."- This paragraph seems to be disjointed. The earlier statement highlights the rate of EBM in Thailand, which followed by ‘little is known about how diabetes affects woman’s confidence…’. Suggest to improve the flow of writing with better connections and relevance between statements. | We edited this section for better flow. Please see Lines 215-219. |
| **Material and Methods** The aims of the study should be placed prior to the methods section. | We made changes as suggested. Please see Lines 224-231. |
| With reference to my prior comment, I highly suggest that the research questions on diabetes self-management and breastfeeding are separated into two different studies. | Thank you for your thoughtful comment. However, our research team strongly believes that we can address four aims in one study.  According to the literature, very little research has been conducted to understand the challenges that pregnant women with T2DM face when establishing breastfeeding and making infant feeding decisions. Therefore, we think that our study is innovative and will provide critically needed insights into where interventions are most needed during and after pregnancy so that Thai women may be effectively supported to meet their breastfeeding goals as they manage their own needs living with T2DM. We appreciate your understanding. |
| **Study Design** "...because we will discover the who, what, and where of diabetes management and breastfeeding experiences by gaining insights from women with T2DM."- Rephrasing needed (grammatical error). | We made changes as suggested. Please see Lines 258-259. |
| Descriptive design? I am not sure if this is a common/usual term to be used to describe a quantitative study design. Do you mean to say cross-sectional study design? | We have edited and now define our study as a cross-sectional descriptive design. See Lines 259-260. |
| **Participant eligibility and recruitment** What if the women are diagnosed with T2DM but lack of experienced in T2DM self-management? Will these participants be eligible? | If pregnant women meet all eligibility criteria, we will recruit them. All participants have been diagnosed with T2DM prior to being enrolled in our study and thus will have had some education and prior diabetes self-management experiences.  Please see Lines 301-303. |
| "Participants will be excluded if they have any significant complications such as blindness or life-threatening illnesses such as myocardial infarction."- Suggest to rephrase to more academically appropriate terms. | We rephrased as suggested.  Please see Lines 303-305. |
| "These interest criteria were derived from the literature focused on pregnant women who will have various experiences on diabetes self-management in pregnancy [23, 24]."- I don’t quite understand this statement. Suggest to rephrase. | We rephrased as suggested.  Please see Lines 310-313. |
| **Ethical considerations** "Participants' rights to participate or withdraw from the study without any effect on care will be emphasized. The PI will give the women a chance to ask questions before deciding to participate in this study."- Redundant phrases as this is typically stated in patient information sheet. | This statement was eliminated. |
| "All participants will receive a signed copy of the consent form, which includes the PI’s study office number. They will be encouraged to call with any questions or concerns and the phone numbers for the offices of human subject protection for any questions they may have regarding their rights as study participants."- With reference to my prior comment, this statement typically stated in the participant information sheet/ consent form, hence not necessary to be elaborated in detailed in the manuscript. | This statement was eliminated. |
| **Sample Size** "...conducting approximately 20 semi-structured interviews"- Do you have any literatures/ references as to why you chose 20 as your approximate sample size? | We added citations as suggested. Please see Lines 356. |
| **Interview Guide** "The interview guide lists open-ended and follow-up questions..."- Need to briefly elaborate the content of the interview guide, what aspects does it cover? | We added more details on how we developed and translated our interview guide from English to Thai. Please see Lines 361-369. |
| **Physiological measures** It is not clear as to the purpose of acquiring these physiological measures as it is not stated as one of the study objectives. | We acquire a woman’s physiological measures as the objective data which will be used for triangulation with subjective data from the interview. Please see Lines 398-399. |
| **Data Collection** "We will give the women a chance to ask questions before deciding whether to participate."- This is a redundant statement as it is not necessary to state in manuscript. | We removed this statement as suggested. |
| **Time 2 data collection** "A mother’s fasting plasma glucose will be measured at the 4-6 weeks postpartum visit."- As per my previous comment, it is not clear as to the indication of this measurement as it is not included in the study objectives. | Thank you very much for your comment. We collected mother’s fasting plasma glucose as one of the objective data which will be used for triangulation with subjective data from the interview. Please see Lines 398-399. |
| **Data management** "All data will be kept in a locked file cabinet or computer behind a locked door."- This may not be necessary to be reported in a protocol. | We removed this statement as suggested. |
| **Quantitative data** What do the authors meant by ‘inconsistent and abnormal values’? | We provided examples of inconsistent and abnormal values. Please see Lines 534-535. |
| **Discussion** Will be good to elaborate further on this, on how the findings of this study may have an impact on patient-centred care. | We added a statement to deepen our response. Please see Lines 585-587. |

We appreciate your thoughtful comments and suggestions. We believe the manuscript has been improved through the revision process. Thank you for considering our revised manuscript for publication.

We look forward to hearing from you.

Sincerely,

Authors
