# Supplementary material for: Self-management of type 2 diabetes mellitus in pregnancy and breastfeeding experiences among women in Thailand: Study protocol
Source: PLoS One. 2023 Jun 12;18(6):e0286646. doi: 10.1371/journal.pone.0286646 (PMC10259789; doi:10.1371/journal.pone.0286646)
Supplement: Attachment — Submitted filename: PONE-D-22-16726 (Post revision Review).docx [file pone.0286646.s006.docx]

Manuscript Review: Self-management of type 2 diabetes mellitus in pregnancy and breastfeeding

experiences among women in Thailand: Study protocol

Journal: Plos One

Journal ID: PONE-D-22-16726

Article Type: Study protocol

| Item | Comments |
| --- | --- |
| General | There still some minor spelling mistake eg ‘compared’ in page 5. Otherwise, most of the issues in the previous review has been addressed by the authors. |
| Title | The title is better after revision and reflects the study protocol |
| Abstract | Clearer study protocol and aim |
| Introduction | Authors have amend the introduction to be much clearer and the flow from one paragraph seems better and easy to follow. |
| Materials and method | **The methodology of the study is explained well and detailed.**  **Data collection**  Page 7- purposive sampling rather than ‘sample’  For physiological measures, why do you need Fasting Blood glucose? Is this to assess the sugar control of the patient? Do you think a 4-point sugar profile will give you better on the patient’s sugar control?  Page 15- spelling ‘entered into ta REDCap database’ |
| Table and images | Table 1- notes  Suggest the author to add ‘EBM’ to the EBF Direct breastfeeding and pumping and bottle. This will avoid confusion by what it meant by exclusive breastfeeding  Figure 1: the heading should be at the bottom of the figure |
| Result | NA |
| Discussion and conclusion | Discussion is more concise in explaining regarding the limitation of the protocol |
| Reference | References are appropriate |
