# Supplementary material for: Self-management of type 2 diabetes mellitus in pregnancy and breastfeeding experiences among women in Thailand: Study protocol
Source: PLoS One. 2023 Jun 12;18(6):e0286646. doi: 10.1371/journal.pone.0286646 (PMC10259789; doi:10.1371/journal.pone.0286646)
Supplement: Attachment — Submitted filename: PONE-D-22-16726R1 Response to Reviewers (5-17-2023).docx [file pone.0286646.s007.docx]

**PONE-D-22-16726R1**

**Self-management of type 2 diabetes mellitus in pregnancy and breastfeeding experiences among women in Thailand: Study protocol**

Dear Reviewers,

We appreciate the opportunity to address your comments and to revise this manuscript. Below please find item-by-item responses to those comments, which are included verbatim. We have indicated all revisions by using text changes, and all page and line numbers refer to locations in the revised manuscript (PONE-D-22-16726.R2). We believe the manuscript has been considerably strengthened with these revisions.

| **Reviewer 1 Comment** | **Author Response** |
| --- | --- |
| Reviewer #1: The authors have addressed most of concern in the first review. There are still minor things to be improved. For further detail please refer attachment. | Thank you for your thoughtful comments. Please see the revised submission in the attached document |
| General: There still some minor spelling mistake eg ‘compared’ in page 5. Otherwise, most of the issues in the previous review has been addressed by the authors. | Please see a revision on Page 5 Line 112. |
| Title: The title is better after revision and reflects the study protocol | Thank you so much. |
| Abstract: Clearer study protocol and aim | Thank you so much. |
| Introduction: Authors have amend the introduction to be much clearer and the flow from one paragraph seems better and easy to follow. | Thank you so much. |
| Materials and method: The methodology of the study is explained well and detailed. | Thank you so much. |
| Data collection: Page 7- purposive sampling rather than ‘sample’ | Please see a revision on Page 7 Line 175. |
| For physiological measures, why do you need Fasting Blood glucose? Is this to assess the sugar control of the patient? Do you think a 4-point sugar profile will give you better on the patient’s sugar control? | We collect fasting plasma glucose (FPG) because it is recommended for assessing glycemic control in early postpartum and essential for creating a future diabetes care plan. On the other hand, a 4-point sugar profile where women collect their self-monitoring of blood glucose 4 times a day is not a usual care practice for all postpartum women with T2DM as it depends on their postpartum FPG level and insulin therapy.  We added a statement on Page 11 Lines 279-281. |
| Page 15- spelling ‘entered into ta REDCap database’ | Please see a revision on Page 15 Line 362. |
| Table and images: Table 1- notes  Suggest the author to add ‘EBM’ to the EBF Direct breastfeeding and pumping and bottle. This will avoid confusion by what it meant by exclusive breastfeeding | We believed you were referring to Table 2. We made a change under Table 2 notes indicating that EBF included direct breastfeeding and expressed breast milk. We decided to spell out 'expressed breast milk' rather than using the abbreviation 'EBM' as we only mentioned it a few times in our manuscript.  Please see revisions on Page 18 Line 425 and Page 13 Line 323. |
| Figure 1: the heading should be at the bottom of the figure | Please see the revised Figure 1 file. |
| Discussion and conclusion: Discussion is more concise in explaining regarding the limitation of the protocol | Thank you so much. |
| Reference: References are appropriate | Thank you so much. We updated the [34] citation from unpublished work to journal article. Please see Page 22 lines 559-561. |

| **Reviewer 2 Comment** | **Author Response** |
| --- | --- |
| Reviewer #2: Overall, the authors have managed to address the feedback/ comments. However, I think the whole manuscript still needs to be proof read for minor grammatical correction. For example. in Line 60 and 62, the author wrote as 'a woman will complete questionnaires', 'a woman will be interviewed', this statements give the impression that only one woman will complete the questionnaire or will be interviewed (instead the authors could modify the statement by stating 'study participants will complete questionnaires', or 'eligible participants will be interviewed..' (suggestion).  Otherwise, the authors managed to revise the manuscript based on the comments and suggestions satisfactorily. | Thank you so much for pointing this out. We made changes as suggested. Please see Page 3 Lines 63-65. |

We appreciate your thoughtful comments and suggestions. We believe the manuscript has been improved through the revision process. Thank you for considering our revised manuscript for publication.

We look forward to hearing from you.

Sincerely,

Authors
